# The State-of-the Art of Environmental Toxicogenomics: Challenges and Perspectives of “Omics” Approaches Directed to Toxicant Mixtures

**DOI:** 10.3390/ijerph16234718

**Published:** 2019-11-26

**Authors:** Carla Martins, Kristian Dreij, Pedro M. Costa

**Affiliations:** 1 UCIBIO—Applied Molecular Biosciences Unit, Departamento de Ciências da Vida, Faculdade de Ciências e Tecnologia da Universidade Nova de Lisboa, 2829-516 Caparica, Portugal; 2 Unit of Biochemical Toxicology, Institute of Environmental Medicine, Karolinska Institutet, Box 210, SE-171 77 Stockholm, Sweden; kristian.dreij@ki.se

**Keywords:** adverse outcome pathways, systems biology, molecular toxicology, co-exposure, environmental risk assessment

## Abstract

The last decade witnessed extraordinary advances in “omics” methods, particularly transcriptomics, proteomics and metabolomics, enabling toxicologists to integrate toxicokinetics and toxicodynamics with mechanistic insights on the mode-of-action of noxious chemicals, single or combined. The toxicology of mixtures is, nonetheless, a most challenging enterprise, especially for environmental toxicologists and ecotoxicologists, who invariably deal with chemical mixtures, many of which contain unknowns. Despite costs and demanding computations, the systems toxicology framework, of which “omics” is a major component, endeavors extracting adverse outcome pathways for complex mixtures. Still, the interplay between the multiple components of gene expression and cell metabolism tends to be overlooked. As an example, the proteome allocates DNA methyltransferases whose altered transcription or loss of function by action of chemicals can have a global impact on gene expression in the cell. On the other hand, chemical insult can produce reactive metabolites and radicals that can intercalate or bind to DNA as well as to enzymes and structural proteins, compromising their activity. These examples illustrate the importance of exploring multiple “omes” and the purpose of “omics” and multi-“omics” for building truly predictive models of hazard and risk. Here we will review the state-of-the-art of toxicogenomics highlighting successes, shortcomings and perspectives for next-generation environmental toxicologists.

## 1. Introduction

Expecting that humans and wildlife are exposed to single toxicants or classes of toxicants is unrealistic. As the basic principle upon which Paracelsus set the foundations for modern toxicology, “it is only the dose which makes a thing a poison”, we must be aware that every biological system is constantly exposed to a multitude of potential hazards whose hazards can be higher than the sum of its parts. Besides very exceptional cases in which pollution (i.e., contamination rising to the point of causing deleterious effects) is caused by a single substance, establishing causal relationships between hazardous substances and deleterious effects to life is one of the toxicologist’s major challenges. Among the few evident single-substance incidents, we recall the Minamata disease caused by methyl mercury poisoning in Japan, traced in the 1950s to industrial charges from a chemical plant [1], and the known associations between occupational exposure to asbestos and human cancer, first disclosed by Doll [2]. Altogether, it is clear that if establishing causality for single substances is already a challenging enterprise, then more so is addressing exposure to two or three. In its turn, dealing with multiple or even countless substances that form part of an organism’s exposome throughout its entire lifespan can seem dystopic. Nonetheless, exposure to mixtures whose toxicants are not entirely (or at all) known a priori is the realistic scenario for occupational toxicologists and environmental toxicologists or ecotoxicologists. In this case, even the classic concepts pertaining to toxicant interactions—additive, antagonist and synergistic—seem out of place. The reader may refer, for instance, to the review by Hernández et al. [3] on pesticide mixtures, for the disambiguation of these terms. 

Developing strategies for the evaluation of risk and safety strategies for chemical mixtures has long been deemed a priority, with many specialized workgroups being constituted in developed countries, arguably first led by the United States and the European Union. However, even the EU’s Environmental Quality Standards (EQSs) for substances in waters, dictated by the Directive 105/EC of 2008, are issued for isolated compounds. In result, a sizable number of strategies have been released in the past decades, with emphasis on statistical procedures, computational tools and bioassay approaches. The reader may refer, for instance, to the works by Allan et al. [4] or Perkins et al. [5]; as well as to the recent review by Kar and Leszczynski [6] on computational approaches to determine the toxicity of mixtures. These range from the traditional quantitative structure–activity relationship (QSAR) models to machine learning. However, these approaches are designed to address known mixtures of toxicants and not the undefinable mixture that characterizes the exposome, human or otherwise. There are, nonetheless, invaluable cases that determined the toxicity thresholds of specific toxicants of classes of toxicants within complex environmental matrices and mixtures of chemicals. It is the case of the sediment quality guidelines (SQGs) derived by Macdonald et al. [7] for major toxicant in marine coastal sediments such as metals, pesticides and polycyclic aromatic hydrocarbons (PAHs), which resulted from empirical models based on vast amounts of chemical and biological data. In essence, these methods were developed to identify toxicological thresholds and not the mechanism. By other words, the majority of approaches to mixtures aim at predicting when they cause harm and not how. This means that effects-directed analysis (EDA) may overlook unknown consequences of exposure, e.g., because they require time to develop (like neoplasms and chronic disease) or because these effects are subtle and not targeted by paradigmatic biomarkers. Nonetheless, EDA and the mode-of-action (MOA) are not self-exclusive. 

The importance of understanding the toxicological MOA underlying exposure to mixtures of chemicals is a paramount component of risk assessment strategies even for regulatory purposes (see for instance Franzellitti et al. [8] and Kienzler et al. [9]). In the first place, toxicodynamics, which aims at disclosing the interaction between chemicals and living systems is positioned at the basis of biomarker discovery (or pattern of biomarkers), which requires solid mechanistic knowledge to ascertain biological plausibility and specificity. In turn, toxicokinetics aims at uptake rates, distribution, metabolization and elimination of noxious chemicals, which unquestionably depends on toxicological mechanism as well. Altogether, both subdisciplines are regarded as integrated elements of the toxic response, which means that knowledge on both is required to determine exposure and predict the probability of harm, i.e., risk. Molecular toxicologists thus aim at fine-tuning their research onto the subcellular targets of contaminants and their mixture and ascertain effects and responses to exposure. The field of science that aims at collecting, analyzing and interpreting data from changes in gene expression and protein activity resulting from exposure to toxicants using high-throughput molecular profiling techniques is commonly termed toxicogenomics (see, e.g., Chen et al. [10] and the classic work by Schmidt [11]).

**Figure 1:**
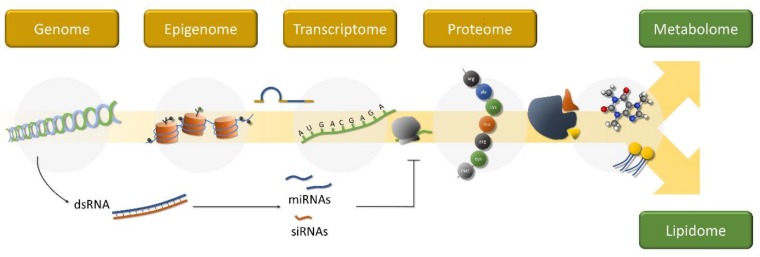
The gene expression path from DNA sequence (upstream) to functional enzymes, yielding concomitant changes to metabolism (downstream). The toxicogenomics approach can focus on any or multiple levels of the molecular cascade than can be impacted by toxicological challenge, either as a response to chemical insult or as an effect. These levels are, nonetheless, two-way interlinked: enzymes are involved every step, from epigenomic modifications and the regulation of transcription and mRNA maturation, for instance. Changes to the proteome will thus affect upstream effects. Bioreactive metabolites can, in their turn, interfere with protein structure and function, as well as with chromatin.

Recent advances in “omics” methods and the computational tools for the analyses of massive amounts of resulting data expanded toxicogenomics from the study of the expression of a few genes of interest toward the quantification of changes in whole-genomes, transcriptomes, proteomes and metabolomes (Figure 1). Naturally, “omics” and the bioinformatics required to analyze such large datasets became a key component of systems toxicology, a derivative of the systems biology perspective that ultimately aims at building truly quantitative and predictive models from the integration of biological data at various levels of organization: from molecule to environment [12]. Under the Systems Toxicology paradigm, toxicogenomics aims at deriving toxicological mechanism and disclosing adverse outcome pathways (AOPs), which are defined as direct associations between specific molecular networks and deleterious effects to biological systems that can be used as signatures of exposure and, therefore, have relevance for risk assessment. By other words, AOPs offer a mechanism-based perspective as an alternative to the classic single-endpoint narrow concept of “biomarker”. Interestingly, the term AOP was first introduced by ecotoxicologists, who quickly adopted “omics” up to the point of becoming pioneers amongst toxicologists, in large part due to the success of these methods on organisms with low or incipient genomic annotation [13]. Besides ecotoxicology, the concepts underlying systems toxicology and “omics” methods are being applied to nearly every subdomain of toxicology to address isolated or combined toxicants, from nanotoxicology to emerging fields such as evolutionary toxicology, the latter of which aims at studying intergenerational exposure to environmental toxicants (see for instance Costa and Fadeel [14] and Oziolor et al. [15]). Even though the relevance of “omics” for studying the toxicology of mixtures has been pointed out years back [16], now is the time to review the subject in face of novel technical achievements, with emphasis on multi-omics approaches, bioinformatics tools, plus their failures and successes in the collusion between traditional and emerging toxicants.

## 2. What Are “Omics”? 

The term “omics” is an informal neologism that applies to various methods designed at the detection, characterization and quantification of large batches of biomolecules in single runs. The essential methods vary according to target, like, for instance, mass spectrometry (MS) for proteins, microarrays and next-generation sequencing (NGS) for mRNAs and gDNAs or nuclear magnetic resonance (NMR) for metabolites. The last decade witnessed a tremendous surge in new or improved “omics” methods and the bioinformatics tools required to analyze the massive outputs, whether these refer to sequences, counts or other forms of quantitative data, and the technical advances appear at an astonishing rate, allowing increased output, sensitiveness and accuracy and low sample input. Nonetheless, validation of findings with single-endpoint methods such as qRT-PCR of a few genes or Western Blot for representative proteins is currently considered good practice when handling “omics”. The reader is then alerted to the need to elect the most adequate quality assessment strategy from current guidelines and available literature.

**Figure 2:**
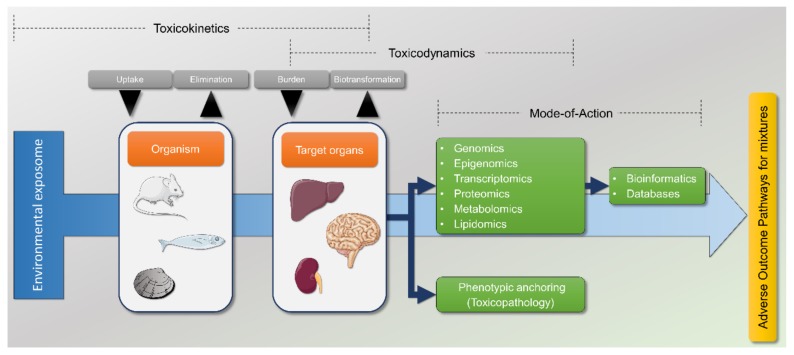
Simplified flowchart linking the environmental exposome with the employment of “omics” methods to address the mode-of-action of toxicants and toxicants mixtures. Mechanism is one of the fundamental steps of the process of disclosing adverse outcome pathways for mixtures, which, in turn, are paramount to develop quantitative and predictive models for environmental risk assessment.

As an illustrative example, in a few years proteomics shifted from one- or two-dimensional gel fractionation procedures coupled to MS–MS detectors for data-dependent acquisition (DDA) to gel independent, highly sensitive data-independent acquisition (DIA) through shotgun methods; selected reaction monitoring (SRM), or sequential window acquisition of all theoretical fragment-ion spectra (SWATH)-MS that can quantify thousands of peptides in highly complex samples (e.g., Simbürger et al. [17], Vidova and Spacil [18]). Similarly, epigenetics expanded from the analysis DNA methylation using, e.g., gene arrays (which are not considered “omics”, though) to methods, like whole-genome bisulfite sequencing (WGBS), which, coupled with NGS, can screen whole-genomes for the reversible epigenetic alterations that modulate expression such as methylation and histone modifications [19]. Toxicologists swiftly adopted these methods and indisputably contributed to their development as the advantages of detecting and quantifying large sets of molecules in single reads from individual samples. This tremendous asset now allows, instead of surveying single or few endpoints that may or not produce clear results, to explore gene and protein sets, for instance, and draw molecular pathways and gene expression signatures resulting from exposure. If properly phenotypic anchoring is provided (e.g., toxicopathology) and toxicodynamics are addressed (as toxicant burdens plus dose- and time-responsiveness), then a link between pathway and effect is established, therefore enabling the determination of AOPs for mixtures (Figure 2).

“Omics” approaches are, nonetheless, challenging in terms of computation and interpretation, technically demanding and highly expensive, which poses serious constraints to experimental design, number of samples and multi-omics approaches. As the technicalities of each “omics” are worthy of a textbook on its own, they will only be briefly explored here. Similarly, we sacrificed providing an extensive list of works for the sake of clarity of take-home messages. Instead, we will focus on specific applications of these approaches to the toxicogenomics of mixtures in environmental toxicology and ecotoxicology following the logical order of events in gene expression: from genome to the products of metabolism and the issue that still tends to be neglected: the interplay between them.

## 3. Genomics and Epigenomics

The genome sets the essential layout for all living organism, which means that minute changes may have a deep impact at all levels of biological organization, from cell to population. Moreover, genotoxicity and mutagenesis have a very significant link with tumorigenesis (see Basu [20] for a recent review). Nonetheless, whole-genome analysis in toxicology is still lagging despite the extraordinary advances in sequencing methods, bioinformatics and machine learning. The high costs of analysis are just a part of the problem. Indeed, reduced or absent genomic annotation of unconventional model organisms and wildlife (unlike murines, zebrafish and humans) is a serious constraint to ecotoxicologists. Interesting prospects arise from state-of-the-art approaches such as Genome-Wide Association Studies (GWAS), which mostly focus on single-nucleotide polymorphisms (SNPs) to detect variants across the genomes of different individuals from the same species or strain. The findings are then matched against phenotypical pathological traits, from resistance to drugs and toxicants to cancer and other diseases (e.g., Giacomini et al. [21]). The approach requires, however, access to batches of sequenced genomes and, so far, has been mostly employed in human toxicity and pharmacology of single substances, with the outcomes being made publicly available in repositories such as the GWAS Catalogue (https://www.ebi.ac.uk/gwas/).

Whereas genotoxicologists were more initially focused on quantifying the amount of DNA damage resulting from exposure to chemicals with direct or indirect genotoxic properties (e.g., adducts and those resulting from hindered repair), there is growing interest in the analysis of polymorphisms either as a consequence of fixed mutations or as indicators of susceptibility to environmental stressors. Despite early promises, either perspective is lagging within toxicology, of mixtures or otherwise. The latter perspective has been proposed for linking human health and environmental stressors, chemicals included, just after the first human full-genome sequencing [22]. This strategy assisted, for instance, the discovery of genes that, in mice, confer resistance to tetrachlorodibenzodioxin (TCDD), a potent mutagen that is an aryl hydrocarbon receptor (Ahr) agonist and therefore able to induce CYP1A expression [23]. The analysis of polymorphisms resulting from exposure to toxicants is acknowledged to be particularly challenging due to limitations in the detection of the low levels of somatic mutations, even by current NGS methods [24] and blocking of polymerases due to DNA lesions. Still, recent advances in NGS sensitivity, error handling and bioinformatics are beginning to produce interesting results, even if not yet linked to the problem of mixed toxicants. Indeed, technical advances and special adaptations of NGS are being developed for the detection of rare mutations and low amounts of DNA damage caused by various agents, chemical included, including Damage-Seq, a sequencing method that immunoprecipitates damaged sites, or Endo-Seq, which circumvents polymerase blocking by forcing strand-breakage at damaged locations (see the recent reviews by Du et al. [25] and Sloan et al. [26]). Additionally, a recent work [27] identified significantly increased incidence of SNPs in the nematode model (*Caenorhabditis elegans*) exposed to Ag nanoparticles in a multigenerational study. Matsumura et al. [28], developed a whole-genome mutation detection procedure in bacteria (*Salmonella typhimurium* strain TA100) as a test to detect the signatures of mutagens, deploying model DNA alkylating agents, namely ethylnitrosourea, methylnitrosourea and ethyl methansulphonate (EMS). These authors disclosed that the assay is able to detect alkylating signatures similar to those registered in human cancer. These findings showed the growing potential of genomic approaches in toxicology, albeit still at their début, in correlation to the advances that are introduced in sequencing technology and computational tools.

Assisted by NGS methods, pyrosequencing (a sequencing method based on the emission of light by the luciferase system during DNA polymerization) and PCR quantification, epigenomics aims at detecting and quantifying epigenetic modifications to the whole genome and identify specific modifications to genes that may compromise their expression. These modifications play a massive role in transcription control by modulating the accessibility to DNA-binding enzymes, which means that epigenetic mechanisms can literally turn on or off certain genes. This must not be confused with simply measuring the total levels of DNA methylation. Although of importance (see for instance Duca et al. [29], for an example on toxicant mixtures, PAHs, in the case, on DNA and RNA total methylation in rats), this strategy does not allow inferring on which genes have their expression inhibited or favored, with consequences for downstream pathways. Methylation of DNA and post-translation histone modifications (forming the “histone code”) are arguably the best-known epigenetic phenomena and are regulated by a battery of enzymes whose function can be modified or compromised by toxicant action (see Marczylo et al. [30], for a review).

Epigenomics has already been applied to the study of toxicant mixtures, in complex matrices inclusively. As an example, Ding et al. [31] discovered differential levels of global DNA methylation between lungs and blood cells in rats exposed in situ to traffic-polluted air for up to seven days. The authors revealed that both increased and decreased methylation occur, which was seemingly gene-specific. In another example, Desaulniers et al. [32] analyzed DNA methylation in the livers of pregnant rats exposed to isolated and mixtures of organochlorine pesticides, methylmercury (meHg) and polychlorinated biphenyls (PCBs), disclosing reduced global genome methylation in the liver, consistent with reduced expression of a methyltransferase. Even though these works have not addressed upstream effects, they illustrate the significance of exploring changes with potentially high impact on gene expression and its regulation. Specifically, they show that epigenetic processes are significantly modulated by exposure to complex mixtures.

## 4. Transcriptomics

**Figure 3:**
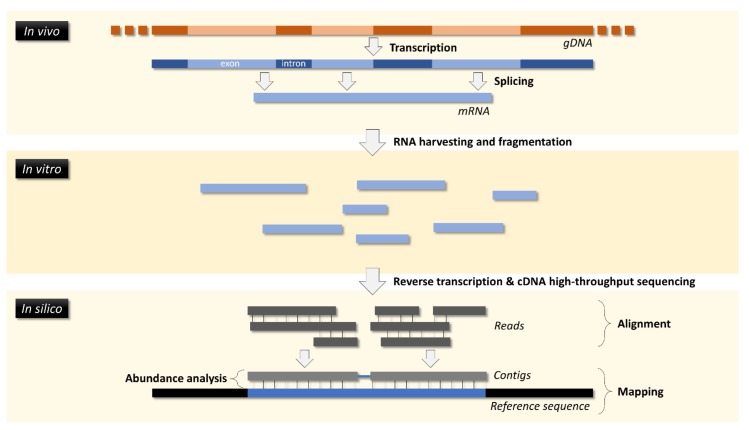
Simplified diagram of the RNA-Seq workflow. The method implies harvesting total RNAs from a biological sample, which is then fragmented, reverse-transcribed to double-stranded cDNA. The cDNA fragments are then sequenced, producing short reads, which are aligned to produce contigs to be mapped against a reference sequence (such as a known transcriptome). The abundance (“counts”) of reads or aligned contigs permits estimating relative expression between experimental conditions.

Transcriptomics addresses global changes to gene expression by targeting mRNAs. Transcriptomics, together with proteomics, is likely one of the most widely employed “omics” by toxicologists. Comparatively to traditional qRT-PCR, transcriptomics methods can target thousands of single mRNAs in a single run, which may render analyses highly cost-effective. The most common methods are microarrays and a form of RNA-directed NGS commonly termed RNA-Seq (Figure 3). It is now clear that the latter, which does not rely on cDNA templates (therefore less dependent of genomic annotation), is effectively able to analyze whole-transcriptomes, generating data on more differentially expressed genes (DEGs), albeit being more challenging to analyze and interpret (refer to Rao et al. [33] for a comparison between methods in a toxicological study with the rat model). Commercial or customized gene arrays for qRT-PCR and suppression subtractive hybridization (SSH) are methods that gained some notoriety within other branches of life science. The first is strictly target-directed but SSH is designed to isolate mRNAs that are presenting in significantly differing number of copies between a control and a treatment, which are then amplified by qRT-PCR and identified by sequencing. As they quantify a reduced number of transcripts (up to a few hundreds) they are a sort of “semi-omics” that is useful and accurate, but not particularly cost-effective. Still, as an example, Evrard et al. [34] used SSHs to study alterations to the transcriptome in the livers of European flounder (*Platichthys flesus*) exposed to mixed herbicides, disclosing alteration related to multiple biological pathways, from immune response to energy metabolism.

Microarrays remain popular in many fields of research, toxicology included, in part due to relatively reduced costs. However, this method requires high genomic annotation because the fundamental principle is to imprint known cDNAs onto a chip, to which fluorescent-labeled cDNAs produced from reverse-transcribed mRNAs of sample will hybridize. Not surprisingly, commercial chips are available for humans, murines, zebrafish, rainbow trout and other standard models. The applications of the method are wide, from single-particle type nanotoxicology to studies involving combined pesticides and PAHs in fish feed, and complex mixtures mimicking crude oil spills, diesel exhausts, on a various range of model organisms (from rats to mussels) using commercial or customized oligonucleotide chips, just to sample a few (e.g., Søfteland et al. [35], Tilton et al. [36], Jensen et al. [37] and Costa et al. [38]). The list of cases is likely too long to be comprehensively presented here but the most important lesson learned is the success of microarrays in complex studies, providing paramount information for risk assessment from toxicological mechanism where traditional limited-endpoint studies fail (see Jensen et al. [37], and references therein). Most importantly, the use of microarrays led toxicologists to use special bioinformatics tools to investigate mechanism, such as gene ontology and gene enrichment-based pathway and network analyses, effectively disclosing or at least contributing to disclose true AOPs for intricate toxicant mixtures. With this respect, we can highlight the works by Zare et al. [39] with the freshwater fish *Pimephales promelas* (the fathead minnow) exposed to mixed endocrine disruptor compounds (EDCs). This was preceded by Lichtensteiger et al. [40], with Wistar rats exposed to complex mixtures of EDCs. In this case, the authors specifically targeted the brain and used an Affymetrix chip designed for the rat brain. Another interesting case study is that of Martínez-Pacheco et al. [41], who exposed a normal mouse fibroblast cell line to mixed metals (Cd, Pb) and As and combined microarray analyses with the expression of miRNAs (determined using a commercial qRT-PCR array). Using the Ingenuity Pathway Analysis (IPA) software suite (provided by Qiagen at https://www.qiagenbioinformatics.com/products/ingenuity-pathway-analysis/), the authors revealed a combination of pathways linked to cell death, inflammation and others. Similarly, Curtis et al. [42], used microarray data from the head kidney of rainbow trout (*Oncorhynchus mykiss*) exposed to complex mixtures of PAHs to analyze susceptibility for infection with the pathogenic bacterium *Aeromonas salmonicida*. These authors retrieved canonical biological pathways from gene enrichment tools [43] using a free (unlike IPA) online software suite called DAVID [44], disclosing de-activation of immune- and infection-related pathways.

The employment of RNA-Seq by environmental toxicologists and ecotoxicologists may be, comparatively to microarrays, in its infancy, but it is already showing its potential for investigating the mechanisms of disease, such as cancer (e.g., Ezerskyte et al. [45]). As it produces far more data than microarrays and is less reliant on annotation, this method can generate even larger data sets from which a more comprehensive list of pathways can be produced. Nonetheless, these same advantages make RNA-Seq data more cumbersome to analyze and interpret. Fortunately, there is a huge effort to adapt statistical procedures to RNA-Seq data, like those available for the open-source software suite R [46], which now accommodates several packages, such as edgeR [47], especially conceived for NGS data, as it does for microarrays. 

Perhaps not surprisingly, RNA-Seq rapidly gained momentum within ecotoxicologists. A recent example comes from the work by Yadetie et al. [48], who employed the method in precision-cut liver slices of the Atlantic cod (*Gadus morhua*) exposed to isolated and binary mixture of the carcinogenic PAH benzo(a)pyrene (B[a]P) and 17α-ethynylestradiol (EE2), revealing putative pathways in which vitellogenin production and the Ahr pathways overlap. These authors also used R for data processing and essential statistics, plus gene enrichment and pathway analysis through DAVID and also the STRING and STICH databases and online tools for surveying gene and protein networks [49,50]. In another example with emerging pollutants, Chen et al. [51] surveyed signaling pathways related to reproduction in female zebrafish recurring exposed to mixed phthalate plasticizers. The authors contrasted DEGs against the canonical pathways in the Kyoto Encyclopedia of Genes and Genomes (KEGG). This is a database that allocates biological pathway data for a large number of species, the zebrafish included due to its acknowledged and expanding value as model organism [52]. Furthermore, physiological condition indices and toxicological traits (e.g., number of viable oocytes) provided important phenotypical anchoring in this same work. To these examples we may add the in vitro study with primary human hepatocytes exposed to insecticides and insect repellents by Mitchell et al. [53], more directed to human risk and therefore better lodged within the domain of environmental toxicology and ecotoxicology, as the previous. These examples illustrate the diversity of research on toxicant mixtures in which RNA-Seq-based transcriptomics has been proving its worth, likely to expand in the near future.

## 5. Proteomics

Proteomics is the study on the proteome, i.e., the set of proteins and peptides that account for structure and metabolic signaling, transport and defense processes in living systems, not excluding cell division and death, differentiation and biomolecule digestion and recycling, for instance. The proteome is essentially the final product of gene expression and, albeit subject to significant interference on all steps that link genes and functional proteins, it can provide a more factual overview of the true metabolic condition of cells and tissues. For disambiguation, the term secretomics applies to a part of proteomics that focuses on secreted proteins. In toxicological studies, proteomics is used to determine how the proteome is modulated by chemical challenge. Proteomics is likely the longest standing “omics” within toxicology and the advances in bioinformatics, plus MS methods that allow higher and more accurate throughput and peptide quantitation, will rightfully guarantee its sustenance in the future. Comparatively to transcriptomics, though, the number of target hits is, traditionally, reduced, which makes it somewhat less cost-effective. In spite of these constraints, the downstream positioning of the proteome in the gene expression cascade and higher independence from genomic annotation than, e.g., microarrays, render proteomics an omics of choice in systems toxicology (see the review by Titz et al. [54]). 

Proteomics has long been widely employed in environmental toxicology and ecotoxicology, of mixtures inclusively. Among the many examples, there are works dealing with unconventional organisms (such as clams and flatfishes) and pollutant complex matrices such as natural estuarine sediments (e.g., Romero-Ruiz et al. [55], Costa et al. [56]). These works are essentially focused on biomonitoring and environmental risk assessment (ERA) strategies. Proteomics is often coupled with more traditional biomarker approaches. Proteomics has, nonetheless, been successfully applied in more mechanistic studies of mixtures, binary or more complex, in many cases in ecologically relevant organisms. It is the example of work with a flatfish (*Solea senegalensis*) exposed to Cd and B[a]P, during which the mechanisms of tissue damage and hindered clearance were investigated by proteomics and phenotypic anchoring with multiple toxicopathological traits, including histopathology [57]. In a similar context but deploying the mussel *Mytilus galloprovincialis* as a model in a work more oriented towards biomarker-search, Maria et al. [58] surveyed changes caused by Cu and B[a]P. Interestingly, among other discoveries, the authors revealed chitin synthase to be de-regulated by mixed and individual toxicants. It must be noted that, in either example above, the bioassays (laboratorial) included co-exposure and isolated substance treatments, as mandated by good practices in mixture assessment. Still, these short-term studies deployed relatively high concentrations of the toxicants to force a noticeable response, which contrasts with the ecological relevance of the two works mentioned in the first place. Demonstrating the importance of modern bioinformatics and available databases in studies involving proteomics, Galland et al. [59] used STRING to demonstrate differential regulation of networks involved in multiple functions, from oxidative stress and metabolite conjugation via glutathione (GSH) recycling to betaine demethylation, in European flounder (*P. flesus*) laboratorially exposed via feed to mixtures of PAHs and PCBs. 

Proteomics has also been applied in studies more focused on potential human health effects. It is the case of Hooven and Baird [60] who exposed MCF-7 cells (human breast cancer) to coal tar and diesel exhaust extracts, as well as to a few individual PAHs. Whereas common mechanisms were found, the authors also concluded that each individual PAH elicits its own pattern of proteome responses. Altogether, these findings sustain the need for caution when establishing causality from exposure to intricate mixtures of pollutants due to potential synergistic effects, i.e., unattainable by individual compounds alone. The zebrafish model, which already joined the ranks of relevant biomedical surrogate models, provides further illustrative examples. Yin et al. [61] exposed wildtype (AB strain) zebrafish (30–90 days post-fertilization juveniles) to mixtures of diketone antibiotics. The authors combined 2DE with matrix-assisted laser time-of-flight MS (MALTI-ToF-MS), validated by qRT-PCR, with toxicopathology to demonstrate the relationship between trace concentrations of the antibiotics with cardiac loss-of-function and damaged structure, providing them paramount toxicopathological validation for their findings.

## 6. Metabolomics and Lipidomics

Comparatively to other “omics” metabolomics and lipidomics are less commonly employed by toxicologists. In a way, both the metabolome and the lipidome can be considered downstream elements of the toxicological mechanisms impacting “omes”, hence being co-joined in a single section. The rising number of works dealing with these “omics” show, nonetheless that technical improvements made in the last few years, including pathway-driven bioinformatics and expanding curated databases for small biomolecules, are steadily convincing toxicologists of their purpose and value.

Metabolomics is the “omics” that embraces identification and quantification of metabolites. The term is often used interchangeably with metabonomics, but the current consensus seems to ascribe metabolomics to the study of endogenous metabolome whereas metabonomics refers to understanding how the metabolome is modulated by external or internal factors, microbiome included (see for instance Robertson [62]). For simplification purposes, we will restrain to the most common term, metabolomics. The work horses of metabolomics are MS and NMR. In mixture toxicology, most studies are based on NMR data, with aquatic animals being the most common biological models, which once again attests how ecotoxicologists are pioneering the use of “omics” in studies whose complexity is augmented by the need to analyze organisms whose physiology is as uncanny and unknown as their genomes. Jordan et al. [63], for instance, used NMR-based metabolomics to investigate the effects of mixtures of EDCs (bisphenol-A, di-(2-ethylhexyl)-phthalate and nonylphenol, all of which are acknowledged emerging pollutants of concern) in the liver and gonads of male goldfish, *Carassius auratus*, exposed via water for 10 days to ecologically relevant concentrations of the toxicants (within the ng/L range). The authors retrieved almost fifty individual metabolites from each organ, matched to KEGG identities and used the IPA suite to disclose a series of modulated intracellular signaling in a synergistic form. Other examples, such as that with the mussel *P. viridis* exposed to Cd and Cu [64] and with *Limnodynastes peronii* tadpoles exposed to mixed pharmaceuticals [65] show the diversity of applications of NMR-based toxicometabolomics. In turn, gas chromatography (GC)–MS metabolomics was used by Xu et al. [66] to identify oxidative damage and disrupted energy metabolism in the liver of rats exposed to the insecticide chlorpyrifos and Cd. Other techniques include nanoflow ultra-performance liquid chromatography-nanoelectrospray ionization-time-of-flight mass spectrometry (nUPLC-nESI-TOFMS), used by David et al. [67] to analyze metabolites in the blood plasma of roach (*Rutilus rutilus*) exposed to wastewater treatment plant effluents contaminated by pharmaceuticals and their metabolites. Tufi et al. [68] also used hydrophilic interaction chromatography (HILIC) coupled with ToF-MS to detect and quantify metabolites in the central nervous system of the freshwater snail *Lymnaea stagnalis* exposed to agricultural surface water extracts containing mixed pesticides and other common pollutants. Spectra were then contrasted against a metabolite database and a large assay of standards for identification. The same authors complemented the MOA approach with endpoints commonly used for EDA such as acetylcholine esterase (AChE) activity and through the identification of target chemicals in extracts, concluding that exposure affected polyamine metabolism, which has been associated to central nervous system disorders caused, e.g., by neonicotinoids.

Lipidomics is a relatively recent subdiscipline that is gaining momentum in high-profile biomedical research, particularly due to the link between metabolic dysfunction and fatty diseases [69], many of which, like hepatic steatosis and phospholipidosis, are familiar to toxico-histopathologists and cytopathologists. Lipidomics, which is currently mostly based on MS and special fractioning for hydrophobic compounds, has yet to conquer its own territory within environmental toxicology and ecotoxicology, related to toxicant mixtures in particular. For these reasons, we are compelled to introduce this important emerging “omics”. An interesting example derives from the application of multiple “omics” to address the problems of mixtures, an issue will be addressed in a section of its own. Phillips et al. [70] studied the toxicity of the main components of electronic cigarettes, as a mixture that among other substances contains nicotine and glycerol, on Sprague-Dawley rats exposed via inhalation and applied transcriptomics, proteomics and a simplified form of lipidomics to detect and quantify the major classes of lipids in blood serum and lung. The authors showed reduced effects resulting from the aerosol exposure, albeit changes at molecular and metabolic levels in rats (liver inclusively). Despite the relatively low throughput from the lipidomics analyses, this work, which combined histopathology and other common endpoints in toxicology, is an interesting example of the systems toxicology flowchart.

## 7. Multi-Omics

Multi-omics (or poly-omics, as often referred to) can circumvent gaps in the toxicological mechanism. Toxicologists must keep in mind that noxious substances can interact differently, yet simultaneously, with a different molecular target. As an example, they can modify the active centre of enzymes involved in pathways as distinct as DNA methylation, repair and transcription; protein folding; lipid peroxidation, etc. These effects and responses, regardless if they are direct or indirect can potentially affect all downstream cell functions, which renders multi-omics an extraordinary tool to disclose a toxicant’s MOA (see for instance Borgert [71]). It is thus reasonable to assume that, when looking for mechanisms in the dark meanders of supra-additive and synergistic consequences of exposure to complex combinations of toxicants, employing methods that can screen various levels is molecular organization is a major asset. The advantages are, however, just as broad as the challenges they pose in terms of cost, experimental and sampling design, interpretation and computation. Having noted this, it is clear that multi-omics is far from routine in environmental toxicology and ecotoxicology of mixtures but the withstanding examples shed light on paths to be trod. A summary of applications is given in Table 1 and discussed below.

As before, important lessons can be learned from ecotoxicologists. For instance, Song et al. [72,73] revealed alterations to the proteome, based on a gel-independent methodology termed isobaric tag for relative and absolute quantitation (iTRAQ) and the metabolome (though NMR) of gills and digestive glands of green-lipped mussel following combined exposure to an organochlorine pesticide and a PAH, hitherto revealing changes at multiple levels: from basal oxidative stress response to cell death and even reproduction. Williams et al. [74] combined microarrays and NMR-based metabolomics to address the effects and responses of exposure to contaminated marine sediments (by recreating a mesocosms in the lab) in European flounder. The authors combined these analyses with environmental chemistry and traditional biomarkers (such as the Comet assay), in an attempt to concatenate mechanism with toxicodynamics and, at least in part, toxicokinetics. This same work, which is yet another good example of the application of the systems toxicology workflow, tracked immune response and apoptosis pathways to be modulated by exposure when genes related to traditional biomarkers failed to respond. Even though much has to be done to disclose an AOP for this mixture and assess its application in predictive toxicology, this work shows that risk assessment of complex mixtures, especially in an environmentally relevant scenario, cannot rely on traditional target-oriented effects.

## 8. Conclusions

Rapid advances in “omics” technologies clearly pushed environmental or ecological risk assessment towards a next generation. As ERA for toxicant mixtures is challenging, particularly if involving naturally complex matrices holding countless noxious substances (whose hazards depend on many factors affecting their bioavailability), it is clear that “omics” are invaluable tools to disclose mechanism and predict effects. We must also highlight the prospects on in silico approaches enabled by machine learning and the ever-expanding public repositories and curated databases for molecules, genes and pathways. As bioinformatics refines towards the ability to build truly predictive models we might expect, in the future, to be able to predict harm to humans and wildlife outside the narrow scope of single-substance environmental quality guidelines or similar thresholds. However, much is there to be done before we can accurately predict the toxicity of mixtures. From lessons learned, we note that “omics” data lacking toxicopathology and some level of toxicokinetics compromise AOP discovery. In addition, toxicologists must acknowledge that each “ome” is most likely able to interfere with another. Consequently, there must be an effort to understand that the exposome is more than the sum of its parts even in the likely scenario that the increasing number of specialists in the field and growing genomic annotation will assist filling-in the gaps towards an integrated perspective on risk assessment.

## Figures and Tables

**Table 1:** Summary of representative applications of multi-omics approaches in the toxicological assessment of mixtures of chemicals (ordered chronologically).

“Omics”	Toxicants	Model	Organ/Tissue	Exposure	Exposure Range	Molecular Alterations	Reference
Transcriptomics (microarray) Metabolomics (NMR, GC-MS)	Ni, Cd, Pb	*Daphnia magna*	Whole-body	96 h	Ni^2+^ (0.5 mg/L), Pb^2+^ (0.5 mg/L), Cd^2+^ (0.05 mg/L)	Genes involved in carbohydrate catabolic processes and proteolysis; genes coding for: mannanase precursor, chymotrypsin-like serine proteases, cellulases, carboxypeptidase, amylase.	Vandenbrouck et al. [75]
Transcriptomics (microarray) Proteomics (2DE, MS)	Imidacloprid, thiacloprid	*Mytilus galloprovincialis*	Digestive gland	4 days	0.1 mg/L; 1 mg/L; 10 mg/L	Protein polymerization; microtubule based movement, and GTPase activity.	Dondero et al. [76]
Transcriptomics (microarray) Metabolomics (NMR)	Wastewater effluents: semi volatile organic compounds	*Mus musculus*	Liver, blood serum and urine	90 days	-	Alterations of lipid, nucleotide, amino acid, and energy metabolism. Disruption of signal transduction processes, hepatotoxicity- and nephrotoxicity-related pathways.	Zhang et al. [77]
Transcriptomics (microarray) Metabolomics (NMR)	Marine sediments: metals, PAHs, organochlorines, butyltins	*Platichthys flesus*	Blood, liver	7 months	-	Xenobiotic metabolism, immune response and apoptosis.	Williams et al. [74]
Transcriptomics (microarray) Metabolomics (NMR) Lipidomics (FT-ICR ^1^ MS)	Benzo(a)pyrene, phenanthrene, Chlorpyrifos, endosulfan	Hepatocytes (*Salmo salar*)	-	24 h	1 µM, 50.5 µM, 100 µM	Suppression of unsaturated fatty acids and steroid biosynthesis. Alterations in linoleic acid metabolism.	Søfteland et al. [35]
Transcriptomics (RNA-seq) Metabolomics (NMR)	Wastewater: PAHs, PAEs, OCCs	*Mus musculus*	Liver and blood serum	90 days	0.1 to 2 ng/L	Molecular pathways related to lipid metabolism and hepatotoxicity	Zhang et al. [78]
Proteomics (2DE, MS/MS) Metabolomics (NMR)	DDT, Benzo(a)pyrene	*Perna viridis*	Gills	7 days	10 μg/L	Impact on of proteins related to oxidative stress, cytoskeleton and cell structure, protein biosynthesis and modification, energy metabolism, cell growth and apoptosis.	Song et al. [72]
Proteomics (RPLC ^1^– MS/MS) Metabolomics (NMR)	DDT, Benzo(a)pyrene	*Perna viridis*	Digestive gland	7 days	10 µg/L	Effects on proteins related to cytoskeleton, gene expression, energy balance, reproduction, development, stress response, signal transduction and apoptosis.	Song et al. [73]
Transcriptomics (microarray) Metabolomics (GC-MS)	(Tri)azoles	Primary hepatocytes (human and rat)	-	24 h	µM range	Activation of pathways related to drug and porphyrin metabolism, peroxisome proliferator-activated receptor (PPAR) signaling pathway and others.	Seeger et al. [79]

^1^ Fourier-transform ion cyclotron resonance, ^2^ Reversed-phase liquid chromatography.

## Abbreviations

2DE	Two-dimensional gel electrophoresis
Ahr	Aryl hydrocarbon receptor
AOP	Adverse outcome pathway
DEG	Differentially expressed gene
EDA	Effects-direct analysis
EDC	Endocrine disruptor compound
ERA	Environmental risk assessment
GC-MS	Gas-chromatography mass spectrometry
GWAS	Genome-wide association studies
KEGG	Kyoto Encyclopedia of Genes and Genomes
MOA	Mode-of-action
MS	Mass spectrometry
NGS	Next-generation sequencing
NMR	Nuclear magnetic resonance
PAH	Polycyclic aromatic hydrocarbon
PCB	Polychlorinated biphenyl
qRT-PCR	Quantitative (real time) reverse-transcription polymerase chain reaction
RNA-Seq	Next-generation whole-transcriptome RNA sequencing
SNP	Single-nucleotide polymorphism.
